# Implementation Strategies and Ergonomic Factors in Robot-assisted Microsurgery

**DOI:** 10.1007/s11701-024-02199-9

**Published:** 2025-01-03

**Authors:** F. Struebing, E. Gazyakan, A. K. Bigdeli, F. H. Vollbach, J. Weigel, U. Kneser, A. Boecker

**Affiliations:** 1 BG Trauma Center Ludwigshafen, Department for Plastic, Hand and Reconstructive Surgery, Department of Plastic Surgery for the Heidelberg University, Ludwig-Guttmann-Straße 13, 67071 Ludwigshafen, Germany; 2 Department for Hand, Plastic and Aesthetic Surgery, Ludwig Maximilian University Munich, Marchioninistraße 15, 81377 Munich, Germany

**Keywords:** Robot-assisted Microsurgery, Robotic Microsurgery, Reconstructive Surgery, Free Flap Reconstruction, Robotic Surgery, Robots in Plastic Surgery

## Abstract

Robot-assisted surgery represents a significant innovation in reconstructive microsurgery, providing enhanced precision and reduced surgeon fatigue. This study examines the integration of robotic assistance in a series of 85 consecutive robot-assisted microsurgical (RAMS) operations. It aims to evaluate changes in the integration of RAMS during the implementation phase in a single institution. The study utilized a prospective database encompassing all robot-assisted microsurgical cases using the Symani surgical system from February until December 2023. A total of 85 robot-assisted operations were analyzed, showing a broad application across various types of reconstructive needs, predominantly in lower extremity repairs (*n* = 41). There were 68 free flap reconstructions (80.0%), ten nerve transfers (11.8%), four targeted muscle reinnervations (TMR; 4.7%), two lymphovenous anastomoses (2.4%) and one arterial reconstruction. The adoption of both traditional and digital exoscopic magnification systems was optimized for each surgical context. The operating room setup and infrastructural challenges for the different anatomic regions are presented. The introduction of robot-assisted surgery entailed overcoming challenges such as adapting to the lack of haptic feedback and navigating ergonomic constraints. Despite these hurdles, including higher operational costs and increased surgery durations, the precision and ergonomic benefits offered by robotic systems may be substantial. Potential solutions and tips to improve the operating times include frequent cleaning of the instruments, active surgical assistance, and rigorous presurgical planning of the logistical setup in the operating room. We showed that there is a preference for the utilization of digital exoscopes over conventional microscopes in RAMS, despite requiring more time per stitch when using the exoscope.

## Introduction

Robot-assisted surgery has emerged as a compelling modality in the field of reconstructive microsurgery, offering potential benefits such as motion scaling for enhanced precision, minimized surgeon fatigue, and an improved ability to perform complex procedures [1]. Since the first application of robot-assisted microsurgery utilizing the DaVinci® platform in 2007 by van Hulst and colleagues, several robotic systems specialized for microsurgical applications have been developed [2]. These systems offer potentially superior ergonomics, the elimination of tremors, and an increased range of movement. However, their application in free tissue transfer and peripheral nerve surgery has remained relatively limited despite their potential to enhance surgical precision and reduce operative morbidity significantly.

The Symani® robotic system consists of two robotic arms that are controlled by the surgeon via two manipulators similar to a pair of forceps. It offers 7 to 20-times motion scaling with the elimination of the physiological tremor. The instruments combined with the micro- and macropositioners provide seven degrees of freedom: X, Y, Z linear motion; roll, pitch, yaw and grip. The system has been utilized in a comparatively small number of cases in lymphatic surgery and free flap surgery [3–5]. Furthermore, it was also used for peripheral nerve surgery [6, 7]. It has also been evaluated for coronary bypass surgery and ophthalmic surgery in an experimental setting [8, 9]. The robotic system differs from the robot used primarily in general surgery, like the DaVinci®, by being designed for a single specialized task—microsurgical anastomosis—rather than performing the entire surgery.

Free tissue transfer is a fundamental component of complex reconstructive surgeries. Despite the routine nature of these procedures, they can be technically challenging and require high levels of microsurgical expertise, particularly in the context of vascular anastomoses [10]. Our key research question is to understand how the integration of a robot-assisted microsurgical system changed during the implementation phase in a single institution and what changes were made to optimize the workflow across multiple reconstructive scenarios.

We have introduced robot-assisted microsurgery into our reconstructive armamentarium over a series of more than 80 cases and over 100 anastomoses and nerve coaptations with the Symani surgical system. In this manuscript, we evaluate our clinical experiences in the implementation of RAMS and aim to provide OR setups for improved workflow.

## Methods

### Data collection

A prospective database was maintained, which included all cases of robot-assisted microsurgery. The clinical cases were not specifically selected to utilize the robotic system; instead, all cases scheduled for the operating theatre with the Symani robotic system were performed using the robot. The study adhered to the Declaration of Helsinki and was approved by the local ethics committee (Medical Commission Rhineland-Palatinate, Mainz, Germany; Protocol number: 2023–16997).

### Surgical technique

**Fig. 1:**
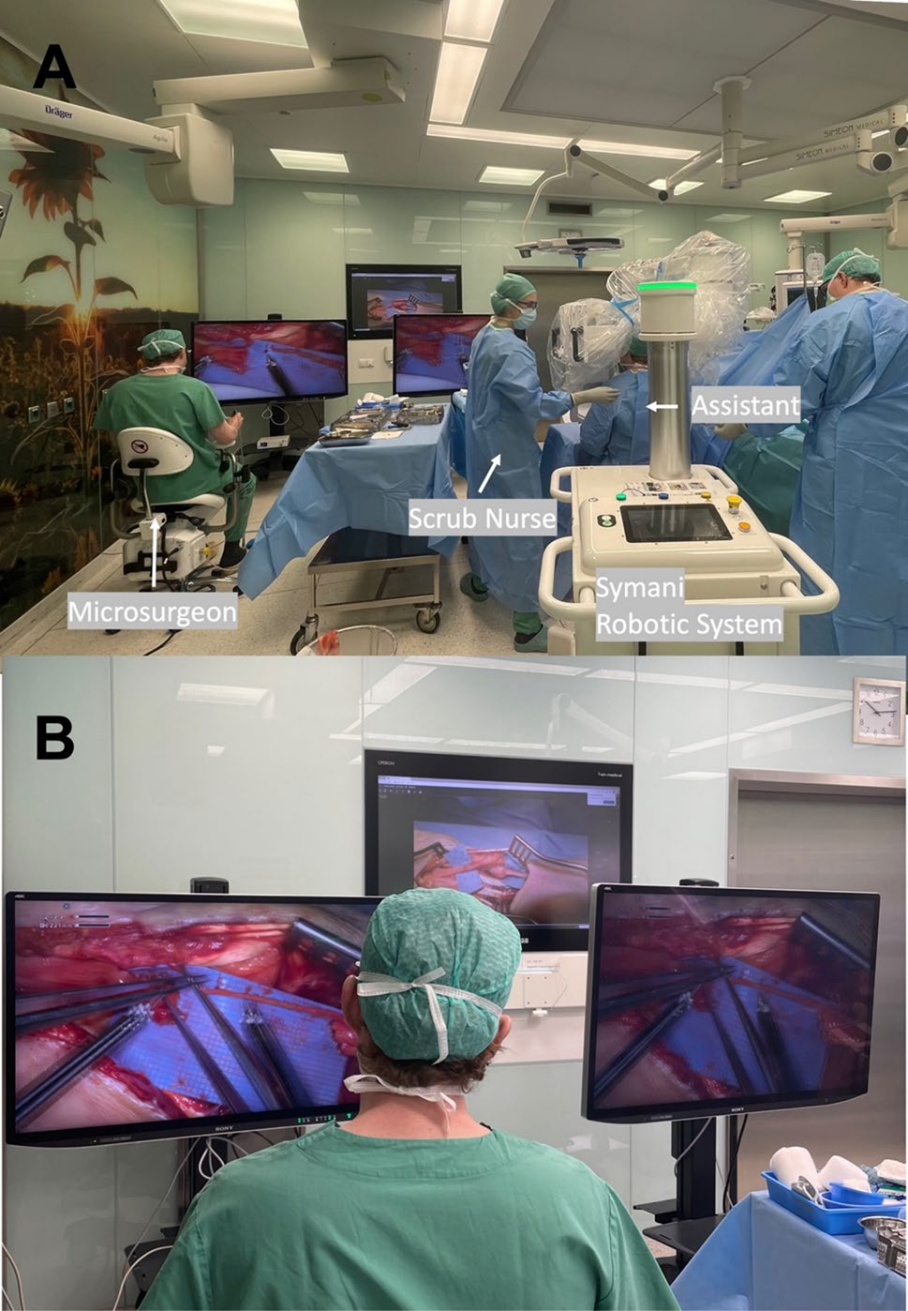
Operating room setup during a nerve reconstruction procedure in the upper extremity. **A** The Symani surgical system is positioned on the side of the reconstruction with the assistant sitting in between the robot arms. The robotic platform is positioned towards the feet of the patient, while the exoscope is coming from the upper side. **B** Using an exoscope, the microsurgeon can sit in a comfortable position, directly in front of a large 3D screen. In this case, the microsurgeon performing the nerve coaptation is not scrubbed in

The free flap reconstructions were performed using standard flap raising techniques as previously described [11]. In each case, one or more anastomoses were performed using the Symani surgical system (Medical Microinstruments, Pisa, Italy). All free flap reconstructions were performed using the regular microinstruments for the Symani system and the super-microsurgical instruments were only used in lymphatic surgery cases. A conventional microscope (Mitaka MM51, Mitaka Kohki Ltd., Tokyo, Japan) or a digital exoscope supported by two 4 K-3D screens (Olympus OrbEye, Olympus K.K., Toyko, Japan) were used for optical magnification. Figure 1 depicts an exemplary setup in the operating room performing a nerve reconstruction procedure in the upper extremity using the exoscope.

### Statistical analysis

We present results as means ± standard deviation (S.D.) or median with interquartile range (IQR). For normally distributed data, statistical analysis was performed using Student’s t-test. Tests including non-normally distributed data were performed using the Mann–Whitney test. Normality was tested with the Kolmogorov–Smirnov test. Significance was defined as *p* < 0.05. Comprehensive data analysis was facilitated using GraphPad Prism Version 10.1.1 for Mac (GraphPad Software, San Diego, CA).

## Results

From February until December 2023, 85 robot-assisted microsurgical operations were performed in our institution. The mean patient age was 53 ± 15 years. There were 55 males and 30 females in the study cohort. The average BMI was 26 ± 4.9 kg/m^2^. The median American Society of Anaesthesiologists Classification (ASA) was two with an interquartile range of one. Arterial hypertension was the most common comorbidity (*n* = 34; 40.0%), followed by tobacco use (*n* = 25, 29.4%) and adiposity (defined as BMI ≥ 30 kg/m^2^, *n* = 15, 17.6%). Table 1 contains information on the patient characteristics.

**Table 1:** Patient characteristics

Parameter	n =85
Age, mean in years ± SD	53 ± 15
Male gender	55 (64.7)
ASA-Classification, Median ± IQR	2 ± 1
Risk factors	
Arterial Hypertension	34 (40.0)
Active tobacco use	25 (29.4)
Adiposity (BMI ≥ 30kg/m^2^)	15 (17.6)
Diabetes	13 (15.3)
PAOD	8 (9.4)
History of thromboembolism	3 (3.5)

Patient characteristics – data presented as n (%), if not mentioned otherwise

**Fig. 2:**
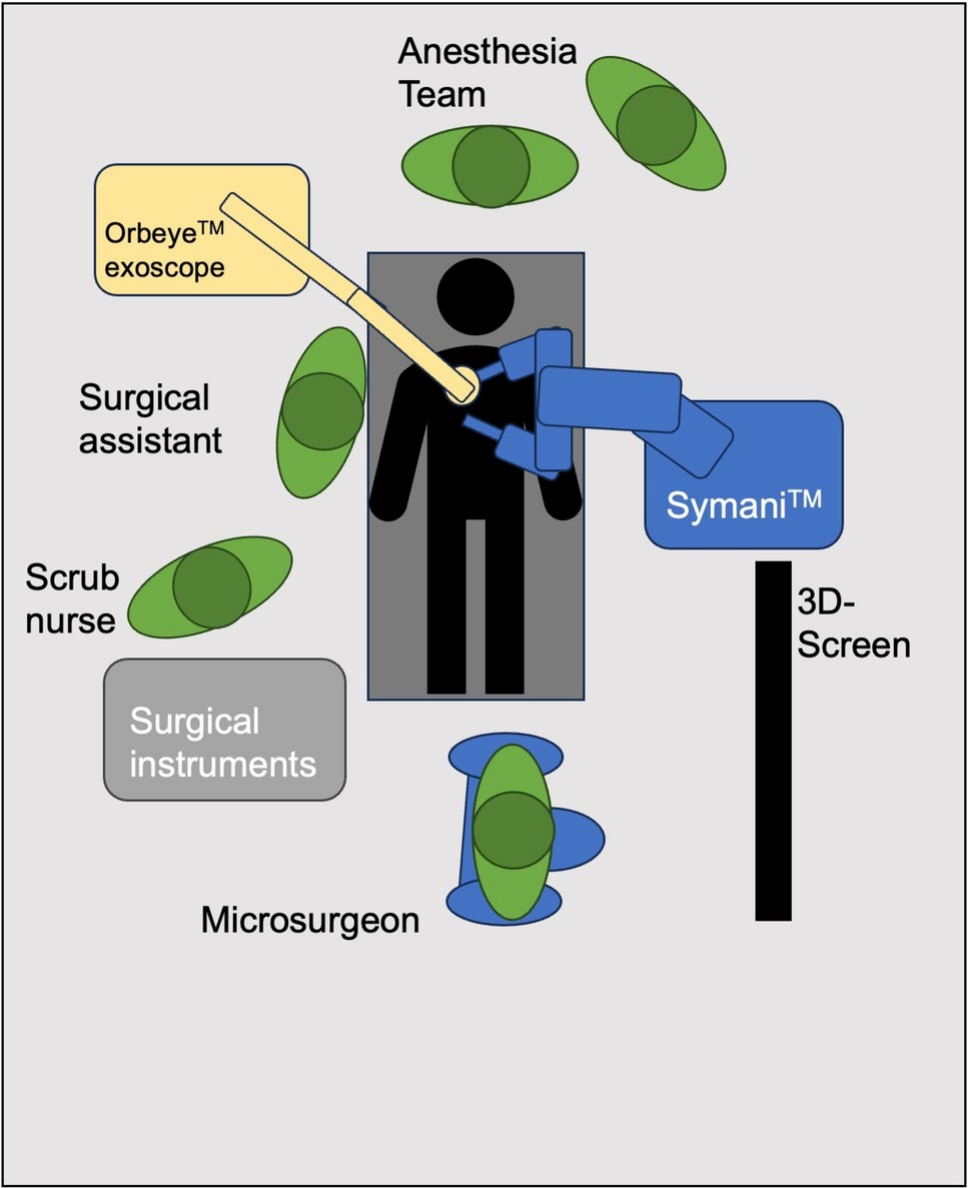
Operating room setup for breast reconstruction. The Symani surgical system is positioned on the side of the reconstructed breast and the assistant on the opposite side. The exoscope is used to enable remote positioning of the operator

**Fig. 3:**
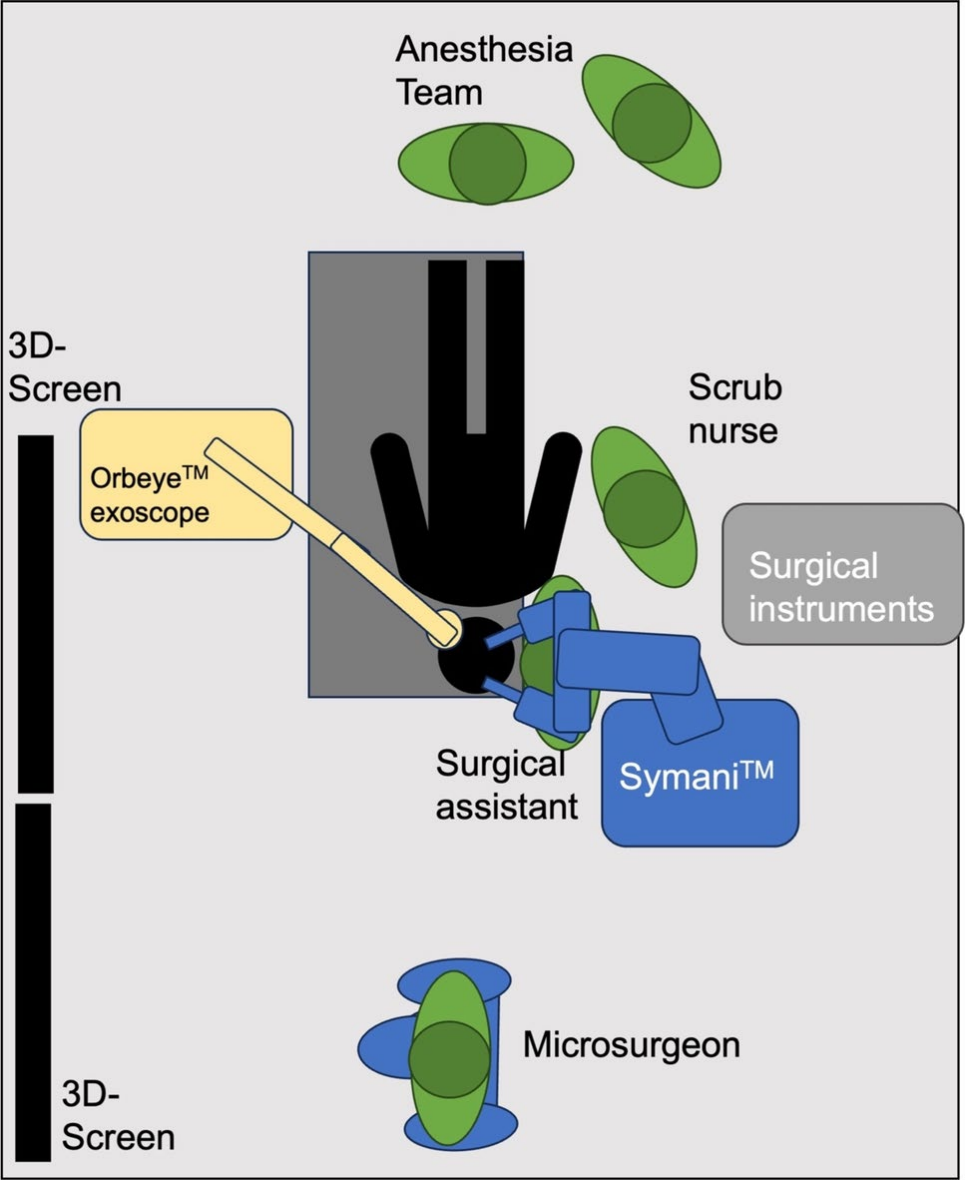
Operating room setup for head and neck reconstruction. The Symani surgical system is positioned on the near side of the reconstruction with the assistant sitting in between the robot arms. The patient is positioned as far back as possible on the table to facilitate easy reach of the robotic instruments. The exoscope is used to enable remote positioning of the operator

**Fig. 4:**
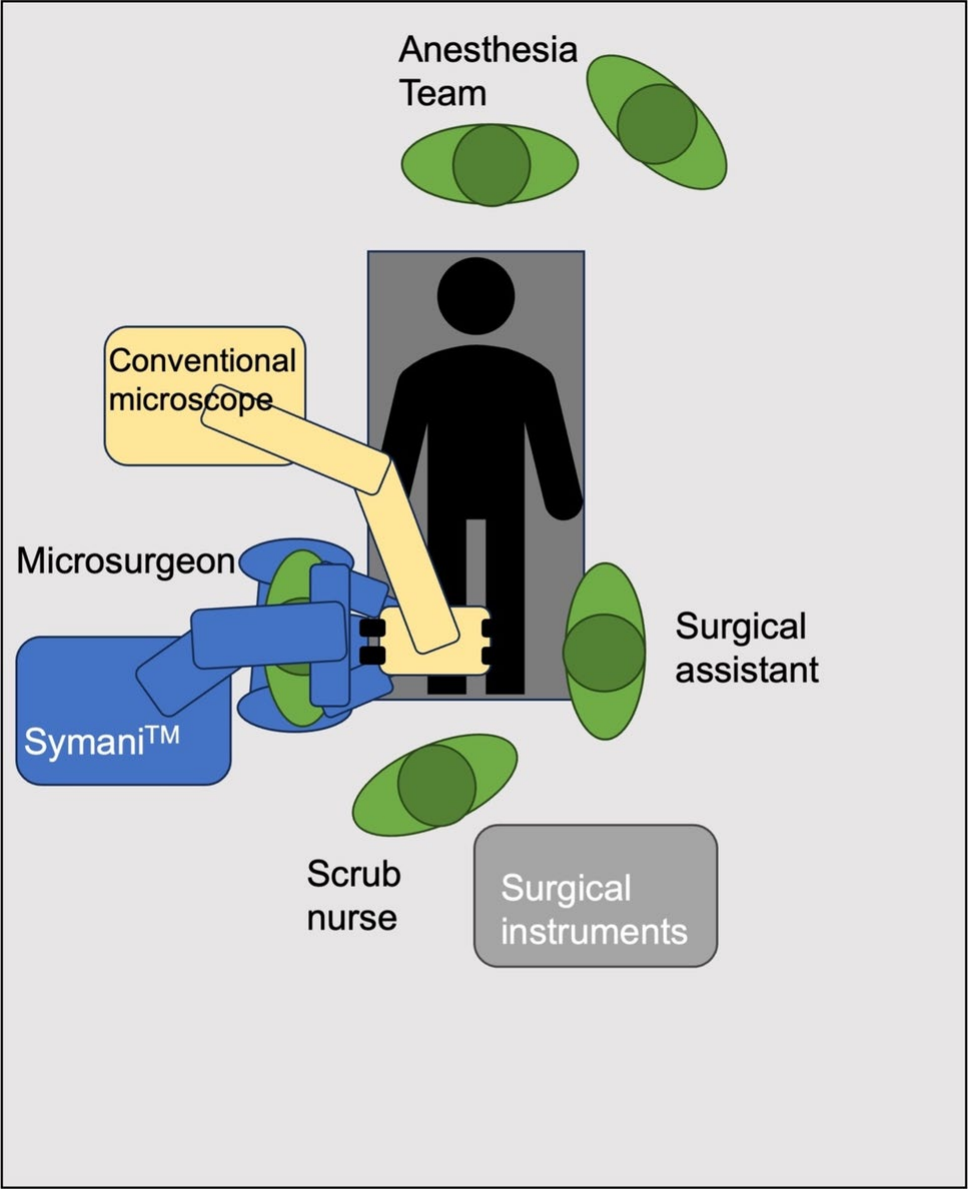
Operating room setup for lymphatic surgery. The Symani surgical system is positioned on the side of the reconstruction with the operator sitting in between the robot arms with the assistant on the opposite side. The microscope and the patient are positioned as far towards the edge of the operating table as possible so that the operator has enough space to manipulate the robotic controllers freely. The necessity for the highest image resolution during supermicrosurgery is best addressed using a conventional operating microscope

**Fig. 5:**
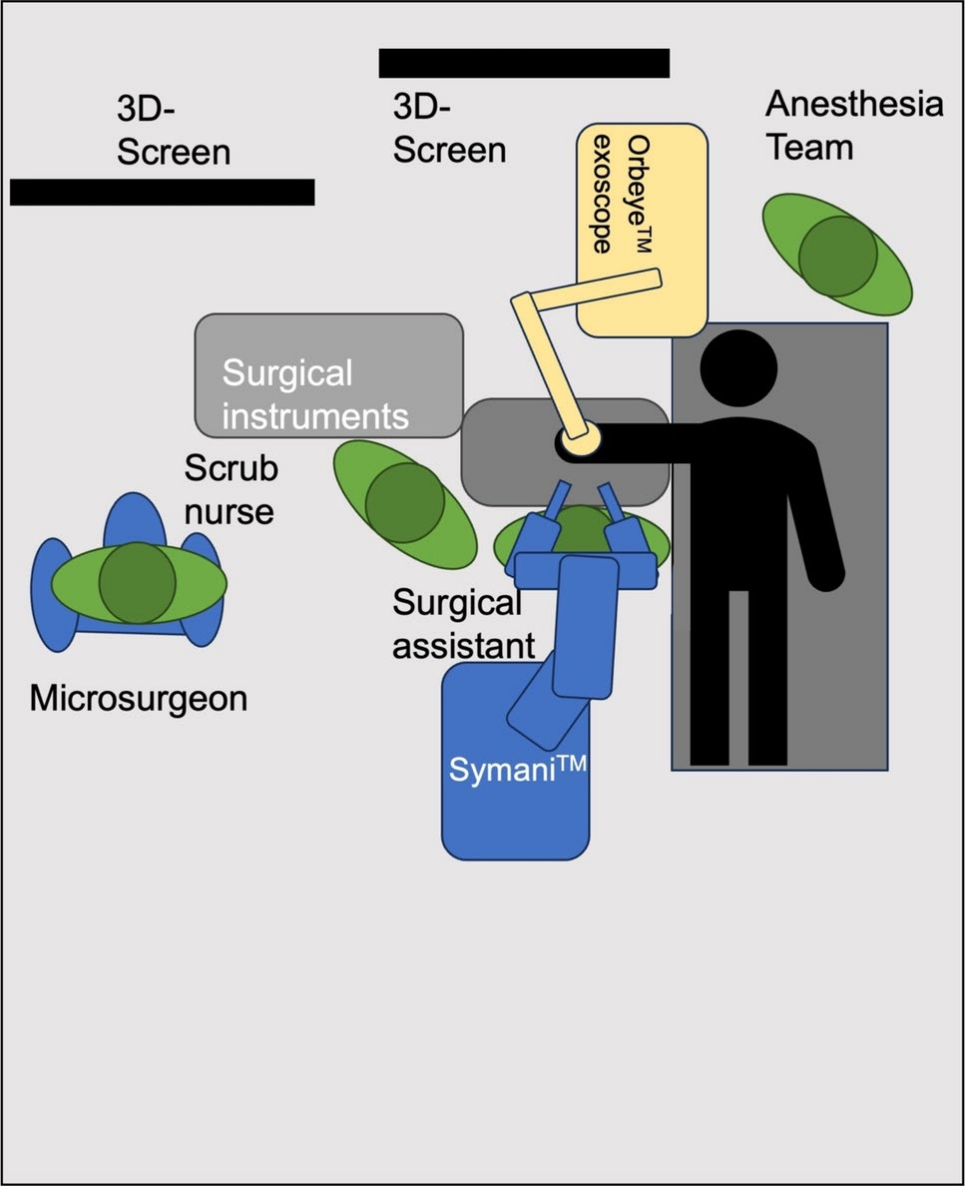
Operating room setup for upper limb reconstruction. The Symani surgical system is positioned on the side of the reconstruction with the assistant sitting in between the robot arms and the assistant on the opposite side. The robotic platform is positioned towards the feet of the patient, while the exoscope is coming from the upper side. Using an exoscope, the microsurgeon can sit in a comfortable position, directly in front of a large 3D screen

Most operations were carried out in the lower extremity (*n* = 42, 49.2%). Thirty surgeries were performed on the upper extremity (35.3%). The rest of the operations were done for breast reconstruction (*n* = 9, 10.5%) and in the head and neck region (*n* = 4, 4.7%). For each anatomic location, an optimized setup of the devices in the operating room was identified and standardized in our institution (Figs. 2, 3, 4, 5).

Free flap reconstructions comprised the largest proportion of cases (*n* = 68, 80%). There were ten cases of nerve transfers (11.7%), four TMRs (4.7%), and two cases of lymphovenous anastomoses (2.4%). In one case, a vein graft was used to reconstruct the ulnar artery (1.2%).

**Fig. 6:**
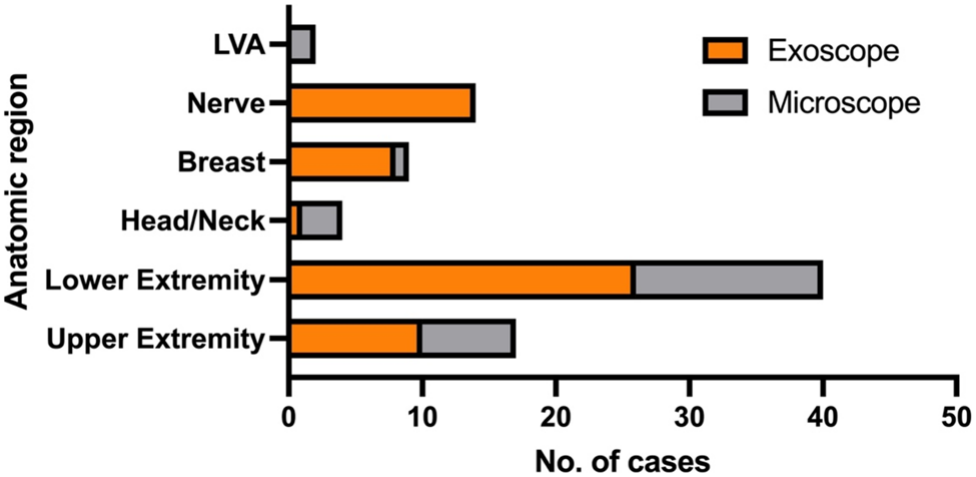
Bar graph depicting the usage of the microscope vs. the exoscope, divided per anatomic region. The lower and upper extremity groups contain only free flap reconstructions

In the 17 upper extremity free flap cases, the microscope and exoscope were utilized at similar rates (*n* = 7, 41.2% and *n* = 10, 58.8%). Among the 40 lower extremity-free flaps, the exoscope was used more frequently (*n* = 26, 65%) than the microscope (*n* = 14, 35%). In a single head/neck case, the exoscope was used (25%), and in three the microscope was chosen (75%). With the exception of one case, all breast reconstructions were performed using the microscope (*n* = 1, 11.1% and *n* = 8, 88.9%, respectively). Figure 5 All 14 nerve cases were done using the exoscope (of those, twelve cases were performed in the upper extremity and two in the lower extremity). Both LVA cases were performed using the microscope. Figure 6 depicts an overview of the utilized optical magnification devices.

**Fig. 7:**
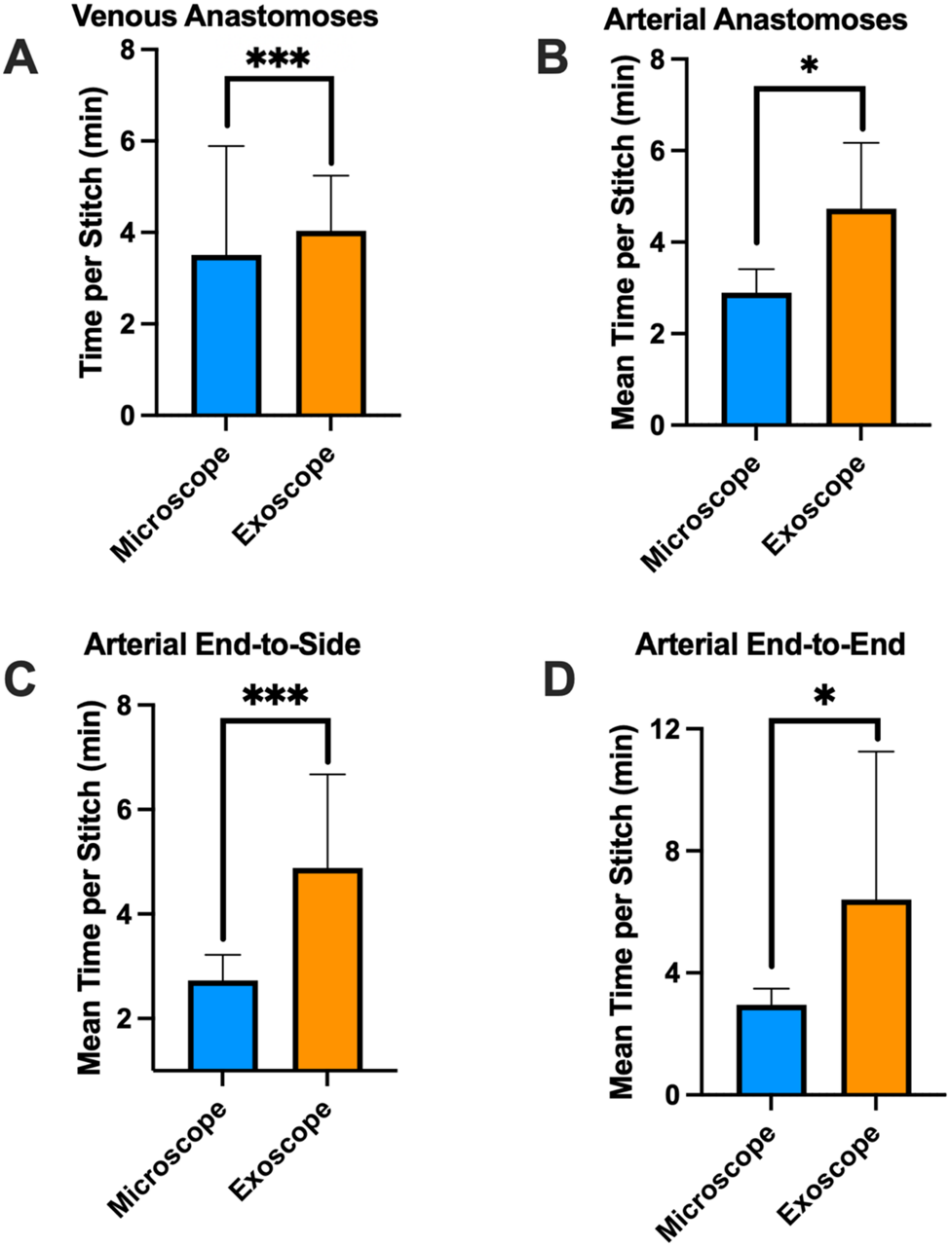
Comparison of general operating times and times per stitch when using the exoscope and microscope. **A** Time per stitch during venous anastomoses. **B** Time per stitch during both types of arterial anastomoses. **C** Time per stitch during end-to-side arterial anastomoses. **D** Time per stitch during end-to-end arterial anastomoses

The mean operating time of the free flaps performed with the microscope was 367 ± 101 min with a range of 183 to 550 min, while flaps that were performed with the exoscope had a mean operating time of 402 ± 99 min with a range of 166 to 568 min. No statistically significant difference was observed when comparing overall operating times (*p* = 0.53). The mean time per stitch during venous anastomoses using the microscope was 3.5 ± 2.4 min, while 4.0 ± 1.2 min when using the exoscope. Performing venous anastomoses with the exoscope required significantly more time per stitch (*p* < 0.001). During arterial end-to-end anastomoses, the mean time per stitch with the microscope (*n* = 10) was 3.0 ± 0.5 min and 6.4 ± 4.9 min when using the exoscope (*n* = 8). In arterial end-to-side anastomoses, the mean time per stitch was 2.7 ± 0.5 min when using the microscope (*n* = 13) and 4.9 ± 1.8 min when the exoscope was used (*n* = 5). Both types of arterial anastomoses required significantly longer times per stitch when performed with the exoscope (*p* < 0.04 and *p* < 0.005, respectively). The mean time per stitch of epineural coaptations using the exoscope (*n* = 17) was 5.0 ± 1.5 min and 2.3 ± 0.8 min when using the microscope (*n* = 6). Epineural coaptations took significantly longer when performed with the exoscope (*p* < 0.001). Figure 7 shows an overview of the times per stitch of the procedures mentioned above.

**Fig. 8:**
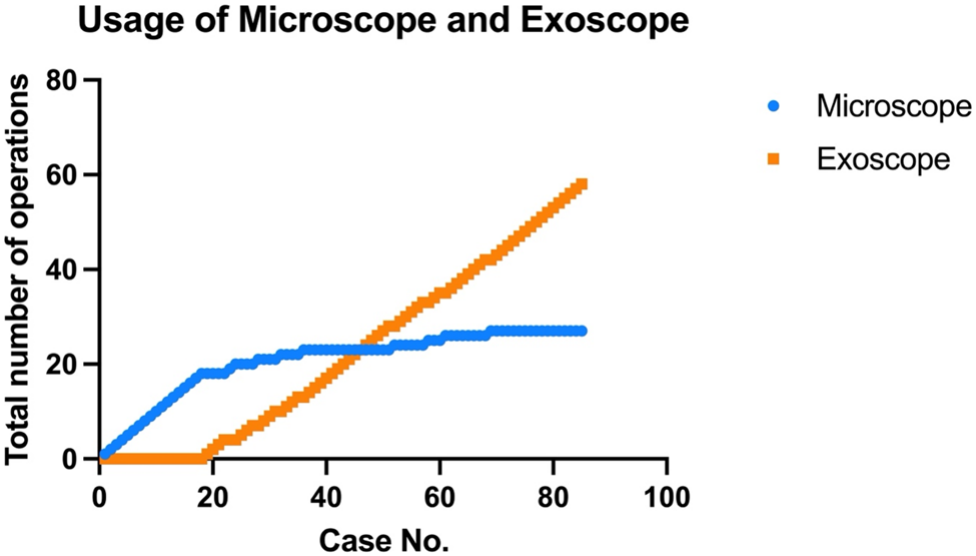
Usage of different optical magnifications over time

Throughout our 85 cases, the 3D exoscope quickly became the primary choice and was used in most cases after its introduction in our department. Figure 8 depicts this evolution in the usage of optical magnification across all included cases.

## Discussion

While robotic-assisted surgery has been widely implemented in the fields of urology, general surgery, and gynecology over the last 25 years, there has historically been a distinct lack of development in the realm of microsurgery [11]. In recent years, however, several robotic systems have been developed specifically for microsurgical applications [12]. Most notably, the MUSA system has been used for lymphatic surgery in a series of clinical cases [13].

In February 2023, we implemented a program for robotic-assisted microsurgery in our department utilizing the Symani surgical system. Since then, we have applied the Symani system in a wide range of reconstructive cases, including extremity defects, scalp reconstruction, lymphatic surgery, and autologous breast reconstruction. With this manuscript, we aim to present our experiences after performing nearly one hundred consecutive cases of robot-assisted microsurgery over a ten-month period with a focus on the different types of magnification systems and logistical considerations necessary for robotic microsurgery.

Traditional microscopes presented operational challenges due to the necessity for the surgeon to be in close proximity to the operating table or patient, thereby restricting the range of movements when using the robotic controllers. Furthermore, the operating table may interfere with the signal transduction between controllers and the robot. These issues were addressed either through meticulous patient and operator positioning or by employing an exoscope which provided superior three-dimensional visualization (Olympus OrbEye, Olympus, Tokyo, Japan). Conventional microscopy offers the advantages of instantaneous image transmission as well as higher contrast and resolution. In our opinion, the superior image resolution makes the microscope the preferred choice for supermicrosurgery. As we show in our data (Fig. 7), the exoscope has replaced it in almost all other scenarios, as the ergonomic benefits are greater, and the provided resolution is sufficient for regular microsurgical applications. With digital exoscopes, the microsurgeon can be positioned freely in the operating room and, therefore, has more freedom of arm and hand movement.

The positioning and setup of the robotic platform alongside the microscope or exoscope require substantial forethought. Improper positioning of the robot or microscope can create ergonomic challenges and potentially impact both the surgical procedure time and the quality of microsurgical outcomes. When implementing an exoscope, particular attention must be paid to ensuring optimal screen visibility, not only for the primary surgeon operating the robotic arms but also for the assistant and scrub nurses. To address this, we acquired a second large screen to improve the visibility for the assistant and nursing staff, instead of the small secondary screen (see Fig. 1).

Throughout our case series, we observed a trend toward increased use of the exoscope following its introduction in our department (see Fig. 7). Today, conventional operating microscopes are used almost exclusively in cases of lymphatic surgery or other supermicrosurgical applications in our institution, where due to the very small vessel sizes, the higher resolution and contrast outweigh the ergonomic benefits of an exoscope, in our opinion (see Fig. 4). In these cases, it is paramount to position the patient and microscope as close as possible to the edge of the operating table. Otherwise, the microsurgeon may not have sufficient space to freely operate the robotic controllers. In a previous study we were able to demonstrate that the use of exoscopes provides significant ergonomic benefits, particularly in anatomical regions subject to the most strain during microsurgical procedures [14]. The results of this study and our clinical practice suggest that surgeon comfort and clinical context drive the choice of magnification system.

Furthermore, using an exoscope comes with its own learning curve and disadvantages [15]. We observed a slight delay in visualization on the 3D screens when using the OrbEye exoscope. Additionally, the surgical team must adapt to the use of the screens with 3D lenses and the overall altered operating room setup. These factors might explain the significantly longer time per stitch when using an exoscope in all types of anastomoses (see Fig. 7).

Even when considering the aforementioned disadvantages of using an exoscope, we found that its ergonomic benefits outweigh these drawbacks in our experience. In an experimental study, Wessel and colleagues analyzed the posture during robot-assisted microsurgery using the rapid entire-body assessment. Their findings demonstrated significantly better ergonomic scores in the group performing robot-assisted microsurgery when compared to the group performing conventional microsurgery, indicating improved ergonomics with robotic assistance [16].

We strongly advocate standardizing the setup of the robotic system and the magnification source according to the specific use case. For instance, our experience indicates that extremity reconstruction necessitates different setup parameters and patient positioning compared to a breast reconstruction. Establishing such standardized protocols before surgery may considerably reduce operating room time and improve ergonomics. We usually position the assistant between the robotic arms and the main operator farther away, using an exoscope (see Figs. 1, 2, 3, and 5). This setup is particularly advantageous in confined surgical sites and if there is a significant step-off of the surgical site, such as when anastomosing to the proximal anterior tibial artery, for example. This way, the assistant does not need to operate with a steep angle of his microsurgical instruments and benefits from much improved ergonomics. In a case series of lymphatic reconstructions, Weinzierl et al. proposed that robot-assisted microsurgery is especially useful in limited and/or deep surgical sites [17].

Throughout our initiation into robot-assisted microsurgery, several challenges were encountered that warrant discussion for the broader surgical community. Similar to the experiences documented by Lindenblatt et al., we also observed specific issues with the robotic system's instruments, notably a tendency for these tools to become sticky after brief periods of use [3]. Our preferred method for maintaining instrument cleanliness involved applying a polyvinyl alcohol wipe two to three times during an anastomosis (Raucocel, Lohmann & Rauscher, Rengsdorf, Germany). A frequent rinse of the instruments with a diluted heparin solution also proved helpful. In conventional microsurgery, the operating room nurse typically cleans the microsurgical instruments during every instrument change. In robot-assisted microsurgery, this task falls to the surgical assistant since the nurse is usually farther away from the robotic instruments, which are fixed in place.

The initial absence of haptic feedback may seem daunting; however, surgeons can rapidly develop visual feedback mechanisms, such as when tying sutures. Consequently, mishandling sutures or applying excessive force to tissues did not pose significant problems in our experience. In contrast to our subjective experience, Beier et al. reported their case series of 23 robot-assisted free flaps and identified the lack of haptic feedback as one of the primary drawbacks, particularly during knot tying [5].

Another paramount hurdle in the introduction of robotic microsurgery are the high purchasing and operational costs of the robotic system, which are currently not reimbursed in the German healthcare system. Although we observed a reduction in anastomosis times over time in our previous study, employing the Symani robotic system still required more time than a conventional anastomosis [10]. This is partially attributable to motion scaling, which slows the surgeon's movements to enhance precision. In a previous study, we analyzed the first fifty cases of robot-assisted microsurgery in our department. We identified a shallow learning curve without a statistically significant improvement in operating times, underscoring the importance of a sustained high caseload [10].

Prolonged surgical times have been reported by nearly all studies exploring robot-assisted microsurgery to date [3, 5, 16–19]. In our cohort, we also found a prolongation of the anastomotic times with arterial end-to-side anastomoses requiring a mean of 37 min [10]. Overall, we did not find a significant difference in overall operating times when comparing cases done using an exoscope and a microscope. In part, these prolonged times have to be expected since the surgeon’s movements are downscaled by 7 to 20 times, thereby slowing overall motion. To improve the anastomotic times, we usually do not use the integrated suture cutter and instead have the assistant cut the sutures. In our experience, the integrated suture cutter is cumbersome and imprecise, as it is integrated into the proximal part of the needle holder. Microscissors that would enable microsurgical dissection are currently unavailable, but their introduction could be beneficial when further preparation of the vessels is needed. Additionally, we have found that having the assistant pass the suture through after the operator places the stitch saves a small but notable amount of time. In general, the assistant plays a far more active and essential role compared to conventional microsurgery. Their tasks include cleaning the robotic instruments, stabilizing the vessel or nerve, and managing the sutures. Furthermore, we advise fully starting up the robotic system so that it is immediately ready after moving it into the operating position. Since the robotic instruments are solely aimed at microsurgical suturing, further dissection of the vessels is not feasible. It is, therefore, essential to prepare both the recipient site and the vascular pedicle thoroughly using loupes before initiating robotic suturing to ensure an efficient and timely anastomosis afterwards.

Evaluating future perspectives of robot-assisted microsurgery in the field of plastic surgery, Henn et al. highlighted the potential of including artificial intelligence (AI) into robot-assisted microsurgery in a narrated review [20]. In the future, AI-driven automation and increased assistance could be used to automatically adjust the motion scaling to the appropriate amount for different tasks, such as making precise needle punctures, passing the suture through or autonomously zooming in and out. Combining the robotic system with a digital exoscope would seem especially beneficial in this scenario. Such integration could enable the unification of controls for both the robotic system and the exoscope, potentially facilitating a smoother and more effortless surgical workflow.

However, even in the face of such potential benefits, we experienced drawbacks. During microsurgical breast reconstruction, we found difficulties due to the respiratory excursion of the thorax during anastomosis. Due to the static position of the robotic instruments relative to the mobile chest wall, the respiratory movements are much more pronounced than during conventional microsurgery, where the microsurgeon rests their hands on the patient, at least partially compensating for the respiratory excursions. In the setting of robot-assisted microsurgery, this issue may be mitigated by adjusting the respirator settings, such as temporarily decreasing the tidal volume, in coordination with the anesthesiologist. In patients with peripheral arterial occlusive disease (PAOD) and heavily calcified arteries, the robot may struggle to penetrate the rigid arterial wall, necessitating conversion to a traditional hand-sewn anastomosis.

While our study adds to the growing body of scientific literature on robot-assisted microsurgery, it is not without limitations. Firstly, there is a relatively low number of cases, especially in the group of lymphatic surgery. Additionally, a rather small group of surgeons performed the surgeries, which may have introduced a performance bias. Furthermore, a randomized study comparing the use of exoscopes and microscopes would have delivered more conclusive results than this retrospective study design.

Despite these challenges, we believe that robot-assisted microsurgery has the potential to revolutionize the field of supermicrosurgery, expanding its accessibility to a broader group of microsurgeons. This may ultimately improve patient outcomes and decrease donor site morbidity, while concurrently reducing the physical strain on surgeons.

## Conclusion

The full integration of RAMS into the clinical routine practice requires careful and standardized OR setups. We showed that there is a preference for the utilization of digital exoscopes over conventional microscopes in RAMS, despite requiring more time per stitch when using the exoscope. Furthermore, we presented OR setups for various reconstructive applications using RAMS. Nonetheless, further research and development are necessary to make robot-assisted microsurgery more widely accessible.

## Data Availability

No datasets were generated or analysed during the current study.

## References

[1] Diana M, Marescaux J (2015) Robotic surgery. Br J Surg 102(2):e15–e28. 10.1002/bjs.9711 25627128 10.1002/bjs.9711

[2] van der Hulst R, Sawor J, Bouvy N (2007) Microvascular anastomosis: is there a role for robotic surgery? J Plast Reconstr Aesthet Surg 60(1):101–102. 10.1016/j.bjps.2006.05.011 17126276 10.1016/j.bjps.2006.05.011

[3] Lindenblatt N, Grünherz L, Wang A et al (2022) Early experience using a new robotic microsurgical system for lymphatic surgery. Plastic Reconstruct Surgery Global Open 10(1):e4013. 10.1097/GOX.0000000000004013 10.1097/GOX.0000000000004013 PMC8747501 35028251

[4] Innocenti M, Malzone G, Menichini G (2023) First-in-human free flap tissue reconstruction using a dedicated microsurgical robotic platform. Plast Reconstr Surg 151(5):1078–1082. 10.1097/prs.0000000000010108 36563175 10.1097/PRS.0000000000010108

[5] Beier JP, Hackenberg S, Boos AM, Modabber A, DuongDinh TA, Hölzle F (2023) First series of free flap reconstruction using a dedicated robotic system in a multidisciplinary microsurgical center. Plastic Reconstruct Surgery Global Open. 11(9):e5240. 10.1097/GOX.0000000000005240 10.1097/GOX.0000000000005240 PMC10482078 37681064

[6] Aman M, Struebing F, Mayrhofer-Schmid M, Harhaus L, Kneser U, Böcker AH (2024) Bionic surgery meets bionic reconstruction–first in-human use of robotic microsurgery in targeted muscle reinnervation. Handchir Mikrochir Plast Chir. 10.1055/a-2241-5678 38513691 10.1055/a-2241-5678

[7] Schäfer B, Bahm J, Beier JP (2023) Nerve transfers using a dedicated microsurgical robotic system. Plastic Reconstruct Surgery Global Open. 11(8):e5192. 10.1097/GOX.0000000000005192 10.1097/GOX.0000000000005192 PMC10424892 37583397

[8] Rusch M, Hoffmann G, Wieker H et al (2024) Evaluation of the MMI Symani® robotic microsurgical system for coronary-bypass anastomoses in a cadaveric porcine model. J Robotic Surg 18(1):168. 10.1007/s11701-024-01921-x 10.1007/s11701-024-01921-x PMC11006781 38598047

[9] Savastano A, Rizzo S (2022) A novel microsurgical robot: preliminary feasibility test in ophthalmic field. Trans Vis Sci Tech 11(8):13. 10.1167/tvst.11.8.13 10.1167/tvst.11.8.13 PMC9400127 35976656

[10] Struebing F, Bigdeli A, Weigel J et al (2024) Robot-assisted microsurgery: lessons learned from 50 consecutive cases. Plastic Reconstruct Surgery Global Open. 12(3):e5685. 10.1097/GOX.0000000000005685 10.1097/GOX.0000000000005685 PMC11213613 38948156

[11] Ghezzi TL, Corleta OC (2016) 30 years of robotic surgery. World J Surg 40(10):2550–2557. 10.1007/s00268-016-3543-9 27177648 10.1007/s00268-016-3543-9

[12] Aitzetmüller MM, Klietz ML, Dermietzel AF, Hirsch T, Kückelhaus M (2022) Robotic-assisted microsurgery and its future in plastic surgery. JCM 11(12):3378. 10.3390/jcm11123378 35743450 10.3390/jcm11123378 PMC9225011

[13] van Mulken TJM, Schols RM, Scharmga AMJ et al (2020) First-in-human robotic supermicrosurgery using a dedicated microsurgical robot for treating breast cancer-related lymphedema: a randomized pilot trial. Nat Commun 11(1):757. 10.1038/s41467-019-14188-w 32047155 10.1038/s41467-019-14188-w PMC7012819

[14] Struebing F, Kneser U, Bigdeli A et al (2024) Ergonomic considerations in robotic-assisted microsurgery. J Craniofac Surg. 10.1097/SCS.0000000000010610 39620987 10.1097/SCS.0000000000010610

[15] Layard Horsfall H, Mao Z, Koh CH et al (2022) Comparative learning curves of microscope versus exoscope: a preclinical randomized crossover noninferiority study. Front Surg 9:920252. 10.3389/fsurg.2022.920252 35903256 10.3389/fsurg.2022.920252 PMC9316615

[16] Wessel KJ, Wendenburg I, Gorji S et al (2023) Combined application of a novel robotic system and exoscope for microsurgical anastomoses: preclinical performance. J Reconstr Microsurg Open 08(2):e88–e96. 10.1055/a-2199-2584

[17] Weinzierl A, Barbon C, Gousopoulos E et al (2023) The benefits of robotic-assisted lymphatic microsurgery in deeper anatomical planes. JPRAS Open. 10.1016/j.jpra.2023.07.001 37546233 10.1016/j.jpra.2023.07.001 PMC10403710

[18] Barbon C, Grünherz L, Uyulmaz S, Giovanoli P, Lindenblatt N (2022) Exploring the learning curve of a new robotic microsurgical system for microsurgery. JPRAS Open 34:126–133. 10.1016/j.jpra.2022.09.002 36304073 10.1016/j.jpra.2022.09.002 PMC9593278

[19] Gousopoulos E, Grünherz L, Giovanoli P, Lindenblatt N (2023) Robotic-assisted microsurgery for lymphedema treatment. Plast Aesthet Res 10:7. 10.20517/2347-9264.2022.101

[20] Henn D, Trotsyuk AA, Barrera JA et al (2023) Robotics in plastic surgery: it’s here. Plast Reconstr Surg 152(1):239–249. 10.1097/PRS.0000000000010270 37382921 10.1097/PRS.0000000000010270

